# The Multiple Impacts of Tropical Forest Fragmentation on Arthropod Biodiversity and on their Patterns of Interactions with Host Plants

**DOI:** 10.1371/journal.pone.0146461

**Published:** 2016-01-05

**Authors:** Julieta Benítez-Malvido, Wesley Dáttilo, Ana Paola Martínez-Falcón, César Durán-Barrón, Jorge Valenzuela, Sara López, Rafael Lombera

**Affiliations:** 1 Laboratorio de Ecología del Hábitat Alterado, Instituto de Investigaciones en Ecosistemas y Sustentabilidad, Universidad Nacional Autónoma de México (UNAM), Morelia, Michoacán, Mexico; 2 Instituto de Ecología AC, Red de Ecoetología, Xalapa, Veracruz, Mexico; 3 Centro de Investigaciones Biológicas, Universidad Autónoma del Estado de Hidalgo, Apartado postal 69–1, 42001 Pachuca, Hidalgo, Mexico; 4 Departamento de Zoología, Instituto de Biología, Universidad Nacional Autónoma de México, A.P. 70–153, México, Distrito Federal C. P., 04510; 5 Instituto de Ecología AC, Red de Ecología Funcional, Xalapa, Veracruz, Mexico; 6 Universidad Intercultural de Chiapas, Unidad Académica Multidisciplinaria Las Margaritas, Chiapas, Mexico; Stanford University, UNITED STATES

## Abstract

Tropical rain forest fragmentation affects biotic interactions in distinct ways. Little is known, however, about how fragmentation affects animal trophic guilds and their patterns of interactions with host plants. In this study, we analyzed changes in biotic interactions in forest fragments by using a multitrophic approach. For this, we classified arthropods associated with *Heliconia aurantiaca* herbs into broad trophic guilds (omnivores, herbivores and predators) and assessed the topological structure of intrapopulation plant-arthropod networks in fragments and continuous forests. Habitat type influenced arthropod species abundance, diversity and composition with greater abundance in fragments but greater diversity in continuous forest. According to trophic guilds, coleopteran herbivores were more abundant in continuous forest and overall omnivores in fragments. Continuous forest showed a greater diversity of interactions than fragments. Only in fragments, however, did the arthropod community associated with *H aurantiaca* show a nested structure, suggesting novel and/or opportunistic host-arthropod associations. Plants, omnivores and predators contributed more to nestedness than herbivores. Therefore, *Heliconia*-arthropod network properties do not appear to be maintained in fragments mainly caused by the decrease of herbivores. Our study contributes to the understanding of the impact of fragmentation on the structure and dynamics of multitrophic arthropod communities associated with a particular plant species of the highly biodiverse tropical forests. Nevertheless, further replication of study sites is needed to strengthen the conclusion that forest fragmentation negatively affects arthropod assemblages.

## Introduction

Habitat loss and fragmentation have been recognized as the most immediate threats to global biodiversity [1,2]. Biotic interactions (e.g. pollination, seed dispersal, predation, etc.) are important forces in structuring biological communities [3,4], and understanding how tropical rain forest fragmentation affects the ways in which organisms interact within and across trophic levels is relevant for conservation studies [4]. The new environments that develop after fragmentation generally fall outside the range of conditions that occurred in the natural forest in which different organisms interact [5,6]. Some novel environments, however, facilitate the colonization of native and/or exotic disturbance-loving species affecting the organization of ecological communities [7–10]. Because ecosystems are conformed by a web of trophic levels, the loss or decrease in the population of one species or functional group could in turn affect species of other trophic levels, thus modifying community structure and dynamics [7,11].

Plants are subject to colonization and/or attack by many different organisms (e.g. viruses, bacteria, fungi, nematodes, insects, among others) that may modify or interrupt their vital functions which affect plant fitness. In spite of this, some other organisms simply use plants as temporal or permanent habitats without damaging them and are even considered beneficial once they can predate on plant pests [12]. In Neotropical forests, antagonistic and mutualistic interactions of some *Heliconia* species (Heliconiaceae: Zingiberales) have been fully described under natural and cultivated conditions [13–15]. Some such organisms are incidental visitors that use heliconias only as a substrate or to search for prey; they are also herbivorous invertebrates feeding on the rhizomes, stems, floral bracts and leaves of heliconias. In contrast, other animal species expend part or their whole lives in the different structures of heliconias. These specialized herbivores include hispine beetles (Chrysomelidae) and caterpillars (Nymphalidae) [13]. Moreover, almost all species of Hispinae beetles are obligate pests of the Zingiberales, including heliconias [16,17].

Therefore, the large number of invertebrates within different trophic guilds (e.g. parasites, saprophytes, herbivores, omnivores and predators) associated with *Heliconia* species provides an excellent model system for understanding the impact of tropical rain forest fragmentation on multitrophic interactions and also arthropod species diversity, abundance, composition, and species turnover. Several types of biotic interactions are increasingly at risk from local and global extinction as a consequence of fragmentation and other anthropogenic disturbances; however, most studies have focused only on species loss and overlook the loss of biotic interactions within different trophic guilds [7,18,19]. Thus, in this study we aimed to understand the patterns and processes underlying the structure of plant and animal communities in a fragmented landscape of southern Mexico. We used as a model system the understory perennial tropical herb *Heliconia aurantiaca* Ghiesbreght ex Lamaire and its associated arthropod fauna. We considered *H aurantiaca* herbs as microhabitats, in which different trophic guilds of arthropods interact (i.e. herbivores, omnivores and predators).

Based on graph theory, several studies have found nonrandom patterns of interactions in plant-animal networks, including for example nestedness and modularity [20–22]. A network is considered nested if species with fewer interactions (peripheral species) are connected with species with the most interactions (central core species) in cohesive subgroups [23, 24]. Alternatively, a modular network indicates that there are subgroups of species that interact more frequently among themselves than with species from other subgroups in the network [22,25]. In fact, network analysis is a valuable tool for studying the diversity of species and interactions within and across trophic levels [19–21].

In tropical forests, studies have shown that although a drastic turnover of animal species from conserved to disturbed habitats the nested structure of ecological networks is maintained [8–10]. Therefore, by using *H*. *aurantiaca* as a model system we evaluated whether or not the structure of ecological networks depends on habitat type and on the trophic guild of the associated arthropod faunas. Specifically we addressed the following questions: (1) Do arthropod abundance and diversity change between continuous forest and fragments? (2) Do trophic guilds change between habitat types? (3) Does individual-based network structure change in *H*. *aurantiaca* plants between continuous forest and forest fragments? (4) What is the contribution of distinct trophic guilds to nestedness in the resulting ecological networks? Because the abundance and composition of invertebrate communities have been shown to differ in human-disturbed landscapes and specialized herbivores decrease therein (e.g. chrysomelid beetles and caterpillars), we expected that fragments and continuous forest do not share similar ecological network structures.

## Methods

### Study area

The Comisión Nacional de Áreas Naturales Protegidas (CONANP), granted permits to work in Montes Azules Biosphere Reserve (MABR) (permission number SGPA/DGVS/07830). The study was conducted within the MABR, Chiapas, in southeastern Mexico (16°06’ N, 90°56’ W, 120 m elev.). The MABR is within the Selva Lacandona region that comprises part of Guatemala and Mexico [26]. Human activities have dramatically reduced the original forested area (500,000 ha) by one-third in 40 years. The MABR contains the majority of the remaining forest of the region (3, 310 km^2^) and constitutes the main component of the Mesoamerican biodiversity hotspot [27,28]. Currently the landscape is composed of a mosaic of land uses including forest fragments, secondary vegetation of various ages, human settlements, cropland, pastures, and paved and unpaved roads. Maximum and minimum annual temperatures are 31.8°C (April-May), and 18°C (January-February), respectively. Annual precipitation is ca. 3, 000 mm [29].

The main vegetation type is lowland tropical rain forest, with trees reaching up to 40 m in height in alluvial terraces along main rivers [30]. There are ca. 4,000 species of vascular plants [26]. Two replicates of contrasting habitat types were considered for this study: old-growth continuous forests and small forest fragments (< 10 ha with 20–30 years of isolation). Tree species richness and density are similar between fragments and continuous forest; however, continuous forest sites hold a greater number of large trees (> 60 cm diameter at breast height) and lower canopy openness than forest fragments [31]. In order to have different populations of *H*. *aurantiaca*, the four study sites were at least 4 km apart from each other and in alluvial terraces [30].

### Study species

Eight species of the genus *Heliconia* have been recorded in the study area, but only three can be found under the shaded conditions of the forest understory: *H*. *aurantiaca*, *H*. *librata*, and *H*. *vaginalis* [32]. The most common of these shade-tolerant herbs within forest fragments is *H*. *aurantiaca*. Herbs of *H*. *aurantiaca* are native to America, and are 0.5–2.0 m tall, with a zingiberoid growth form and a spiral bract arrangement with erect inflorescences [32].

The foliage of *H*. *aurantiaca* is attacked by three major groups of insects [33]: (i) hispine beetles—mostly small chrysomelids, some specialized in the genus *Heliconia* [16, 17,34]; (ii) caterpillars represented by a broad gradient of body size, including those large and specialized *Caligo* and *Opsiphanes* (owl butterflies) [33,35]; and (iii) leaf-cutter ants of the genera *Atta* and *Acromyrmex*—which are some of the most generalist herbivores in the Neotropics [36].

Preliminary observations in the study area suggest that the density of specialist hispines declines in forest fragments, whereas that of leaf-cutter ants increases. No change has been observed in the abundance of caterpillars [37]. Three species of hispine beetles (i.e. *Cephaloleia* spp) have been recorded feeding on the foliage of *H*. *aurantiaca*; one species belongs to the belti-complex, and the other two species belong to the instabilis-stenosoma complex. In addition, six species of caterpillars have been observed feeding on *H*. *aurantiaca* foliage; three of which are considered specialized in Zingiberales (i.e. *Caligo uranus*, *C*. *memnon* and *Opsiphanes tamarindi tamarindi*), and the rest are considered as generalist herbivores (i.e. *Tarchon felderi*, *Antichloris* sp., *Acharia* cf. *stimulea* [37]). Furthermore, the numbers of leaf-cutter ant nests (*Atta cephalotes*) are higher in fragments than in continuous forest [37]. Other invertebrates found to inhabit different structures of *H*. *aurantiaca* foliage include spiders, harvestmen, other ants, other beetles, bees, cockroaches, ticks, flies, mosquitoes, grasshoppers, phasmids, bugs and snails (Benítez-Malvido, unpublished data). The density of and the physical and chemical foliage traits of *H*. *aurantiaca* (i.e. leaf area, leaf toughness, leaf condensed tannins, number of shoots per plant, etc.) have been shown not to differ significantly between continuous forest and forest fragments (Table 1).

**Table 1:** Characteristics (mean ± SE) of *Heliconia aurantiaca* plants in continuous forest and forest fragments in southeastern Mexico (modified after [37]; Santos & Benítez-Malvido, unpublished data).

	Continuous Forest	Forest Fragments
Host density (clumps/314 m^2^)	98 ± 13	137 ± 22
No. of leaves	134 ± 18	154 ± 14
Leaf area (cm^2^)^a^	1532 ± 19	1396 ± 39
No. of shoots per year ^a^	38 ± 04	43 ± 09
No. of flowers per year ^a^	143 ± 20	172 ± 21
Absorbance of condensed tannins	26 ± 02	25 ± 032
Leaf toughness (g/cm^2^)	2626 ± 242	2738± 145

^a^Mean leaf area, shoot number and flower number were calculated from 18 plants per habitat

### Invertebrate sampling

To assess whether or not forest fragmentation affects local arthropod diversity and abundance within different trophic guilds, in each habitat at each study site we randomly selected seven individuals of *H*. *aurantiaca*. Individuals of *H*. *aurantiaca* were at least 5 m apart from each other on all study sites and habitat types. Arthropods were collected in three surveys during May, August and November of 2013. In each survey, we randomly selected new individuals, and therefore no plant of *H*. *aurantiaca* was sampled more than once. In total, we sampled 84 different plants: 42 in continuous forest and 42 in forest fragments. The samples of one plant per habitat were lost however.

We collected all arthropods (mainly adults and a few larvae) found on *H*. *aurantiaca* clumps. Firstly, we made a visual inspection of the plant to catch jumping insects and spiders. Secondly, we collected arthropods by tapping the plant with a stick while holding a collecting tray underneath. Finally, we carefully reviewed all plant structures including leaf blade, petiole, and bracts to collect all the arthropods in the individual clumps. Arthropods were placed into plastic pots containing alcohol 70% and subsequently identified to the lowest possible taxonomic level. Voucher adult specimens were deposited at the Instituto de Biología and at the Instituto de Investigaciones en Ecosistemas y Sustentabilidad collections, UNAM, Mexico. Some of the collected specimens might be undescribed or new records for Mexico. Although caterpillars (Lepidoptera) have been reported as pests of *Heliconia* elsewhere [15], we did not collect their larvae in our surveys [33]. All collected arthropods were subsequently classified into four broad trophic levels as follows: herbivores, omnivores, scavengers and predators [12,15]. The designation of the trophic guilds was based on the literature, morphological examination of the mouth structures and on expert knowledge. Scavengers were poorly represented (three species of Coleoptera, Ptinidae and eight individuals) and therefore were excluded from further analysis (S1 Table). Insect herbivores can feed either externally or internally within plants (e.g., chewing insects, leaf miners, fluid and/or phloem feeders, and gall-making insects) [15]. However, because of the limited knowledge on the arthropod community in the study region, it was only possible to classify insects and other arthropods into the above trophic guilds regardless of their feeding mode.

### The arthropod community

To detect differences in arthropod density within trophic guilds between habitats we used generalized linear models (GLM) for count data with a Poisson error distribution and a logit link function; we also checked for overdispersion [38]. Preliminary analysis showed that arthropod density did not differ between sampling times (χ^2^ = 4.05, df = 2, *P* = 0.1) and therefore, data from the three surveys were pooled for all analyses as indicated for repeated measurements [39, 40]. Sample coverage and true diversity metrics were calculated in SPADE [41]. Generalized linear models and bootstrap plot curves were carried out using R. 3.0.1 [38,42]. Completeness of arthropod species inventory per habitat type was evaluated as the percentage of observed species in relation to the number of species predicted by the sample coverage estimator, which is the least biased estimator of sample completeness [43]:

$$Cn=1-\frac{f1}{n}\left|\frac{\left(n-1\right)f1}{\left(n-1\right)f1+2f2}\right|$$

where *f*_*1*_ and *f*_*2*_ are the number of singletons and doubletons in the sample, respectively, and *n* is the number of individuals. Furthermore, in order to make direct statistical comparisons of species richness between habitat conditions, we generated individual-based rarefaction curves by using the on-line resource ¡NEXT [44] that is based on R-statistical language.

We calculated the number of effective species by using the measure of true diversity of Order 1 [45], which weights each species exactly by its frequency in each habitat type (i.e. favoring neither rare nor common species). Given the presence of rare species (singletons) in both habitats, we calculated the estimated diversity by employing the Chao and Shen method [46], which is a non-parametric approach that allows for an accurate estimation when there are unseen species in a community. In addition we calculated the diversity Order 2 (inverse of the Simpson index) [45], by using the MVUE estimator (minimum variance unbiased estimator). Finally, to assess the turnover of species composition between habitats we used similarity Bray-Curtis index between the two habitats [47], and differences were analyzed with a permutational multivariate analysis of variance after 999 permutations [48].

### Patterns of species interactions by using an interaction network approach

Firstly, we included the presence of different animal species visiting the 82 different individuals of *H*. *aurantiaca* in each habitat (continuous forest and forest fragments) as independent interaction networks. Each individual-based animal-plant network was built by an adjacency matrix ***A***, where *a*_*ij*_ = number of interactions from an individual plant *j* by the animal species *i*, and zero otherwise. We then calculated the diversity of interactions (*DI*) for each network. This metric is derived from the Shannon’s diversity index and ranges from 0 (no diversity) to infinity [49]. We used the *NODF* metric (based on overlap and decreasing fill) [50] in the ANINHADO program [51] to calculate if selective species would visit only a subset of plant individuals visited by the central core species. We choose this metric because it is less prone to Type-I statistical error: in other words, the incorrect rejection of a true null hypothesis (a “false positive”). The *NODF* metric reduces the chance of overestimating the degrees of nestedness in ecological networks [50]. To assess if the nestedness value observed was higher than expected by random interaction patterns, we tested the nestedness of each network with 1000 networks generated by Null Model II. In this null model, the probability of occurrence of a new interaction is proportional to the number of interactions of a given species [20]. Once our interaction network in the forest fragments was significantly nested (see the Results section), we explored whether the three trophic guilds (herbivores, omnivores and predators) contributed equally to the structuring of the nested pattern. We estimated the degree to which the interactions of plants or trophic guilds increase or decrease the network’s overall nestedness (contribution to nestedness, *cn*) [52]. This estimate is derived from the *NODF* metric, and positive values of *cn* indicate a higher contribution to the nested structure. Thereafter, we used a one-way ANOVA followed by a Tukey post hoc test to test for differences in mean values of *cn*_*i*_ among the three trophic guilds and plants.

## Results

### Fragmentation and arthropod abundance

Overall, we collected a total of 727 individuals, 285 (71 morphospecies) in continuous forest and 442 (55 morphospecies) in forest fragments. Arthropods collected from *H*. *aurantiaca* herbs included five orders and 27 families: Araneae, Coleoptera, Hemiptera, Hymenoptera and Orthoptera. Hymenoptera showed the greatest abundance: 470 individuals, all of which were ant species (Formicidae, S1 Table). All of the 82 sampled *H*. *aurantiaca* plants were occupied by arthropods; however, not all arthropod taxa were present in all plants. The number of arthropods per plant ranged from 1 to 25 individuals in continuous forest and from 1 to 55 individuals in forest fragments.

**Fig 1:**
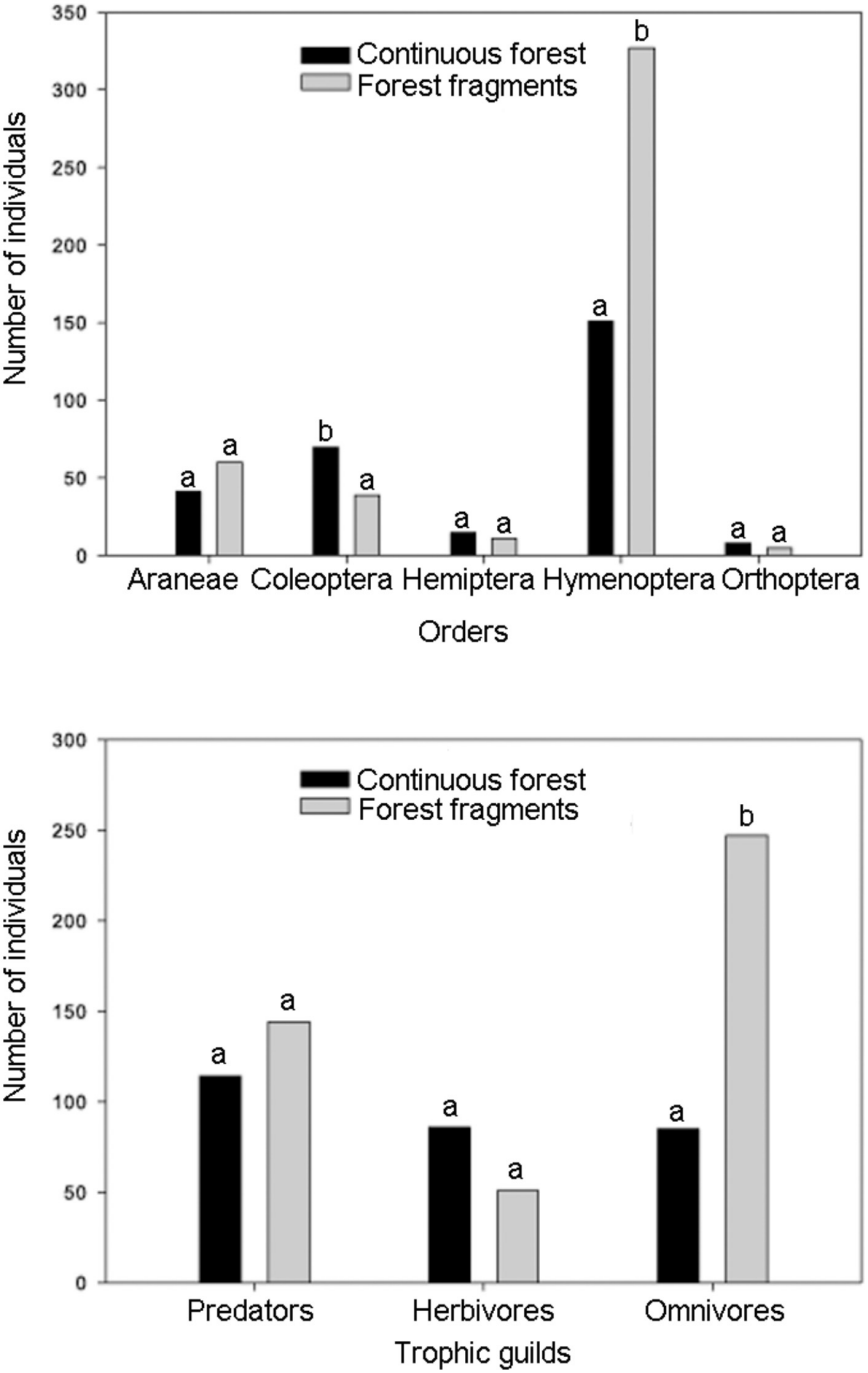
Total number of individuals within the five most abundant arthropod taxa and trophic-guilds inhabiting *Heliconia aurantiaca* herbs in continuous forest and forest fragments in southeastern Mexico. The number of arthropods was obtained from 41 clumps of heliconias per habitat type.

Arthropod abundance differed significantly between habitats (χ^2^ = 34.14, df = 1, *P* < 0001), with forest fragments having the greatest abundance. Furthermore, there were significant differences in the density of arthropods within taxonomic orders between habitats (χ^2^ = 392.42, df = 4, *P* < 0001). Forest fragments hold a significantly greater abundance of Hymenoptera (ants) than continuous forest, whereas continuous forest holds a greater abundance of Coleoptera (beetles) than forest fragments (Fig 1).

### Forest fragmentation and trophic guilds

The arthropod community associated with *H*. *aurantiaca* in continuous forest and fragments was grouped into three distinct trophic guilds: omnivores, herbivores and predators, with ant, beetle and spider species being the most representative taxa of each guild, respectively (S1 Table). The most common herbivore species were tortoise beetles (Chrysomelidae: Cassidinae) and bugs (Hemiptera); the most common predators were jumping spiders (Salticidae), whereas the most common omnivores were ants of the genus *Pheidole* and *Pseudomyrmex* (Formicidae). The abundance of trophic guilds differed significantly between habitats (χ^2^ = 161.29, df = 2, *P* < 0.001), with omnivore species being significantly more abundant in forest fragments than in continuous forests (Fig 1). The number of species per trophic guild in continuous forest and fragments was as follows: 37 and 43 for predators; 15 and 18, herbivores; and 3 and 10, omnivores, respectively.

### Fragmentation and arthropod diversity

**Fig 2:**
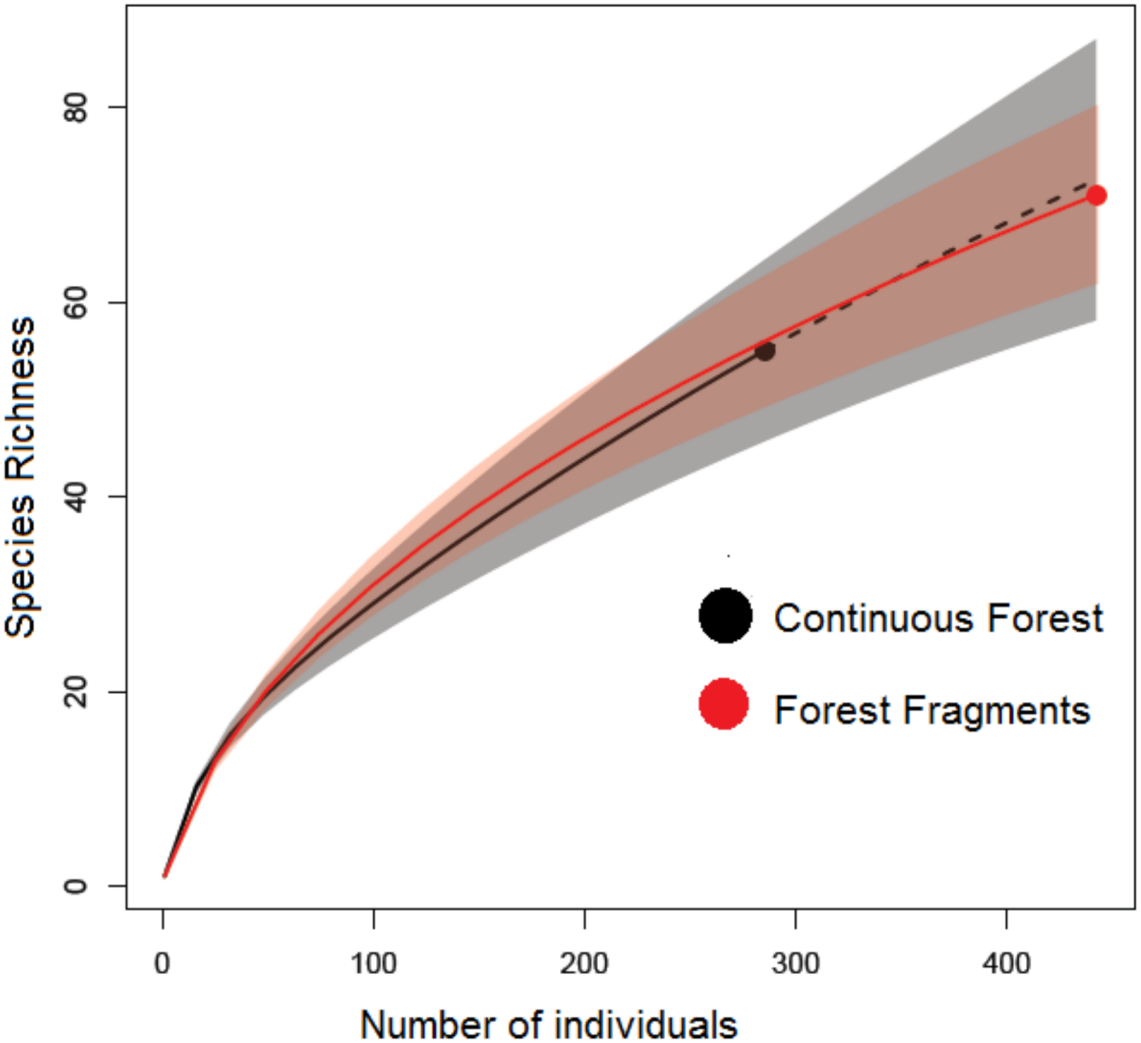
Rarefaction curves of species richness (bootstrap method) based on the number of arthropods inhabiting *Heliconia aurantiaca* herbs in continuous forest and forest fragments in southeastern Mexico. The shaded area represents the confidence intervals of 95% based on 1000 randomizations.

Although estimations of sample coverage were high for both habitat types, 87% for continuous forest and 91% for forest fragments, none of the rarefaction curves in either habitat reached an asymptote (Fig 2). Comparing rarefaction curves at the lowest abundance value of 285 individuals, species richness showed no significant differences between habitats; and comparing species frequencies according to diversity Order 1, there were almost the same effective species in continuous forest (25.83) as in forest fragments (22.54). In contrast, with diversity Order 2, diversity was two times greater in continuous forest (12.48 effective species) than in forest fragments (6.96 effective species). Finally, the permutational multivariate analysis of variance showed significantly different species composition (Bray-Curtis similarity index) between habitat types (pseudo-*F*_1, 2_ = 1.73, *P* = 0.05). Some species were exclusively found in continuous forests (e.g. *Dolichoderus lutosus*) whereas others in fragments (e.g. *Camponotus planatus*) and some others were common in either habitat (e.g. *Dolichoderus bispinosus*, see S1 Table).

### Fragmentation and the structure of ecological networks

**Fig 3:**
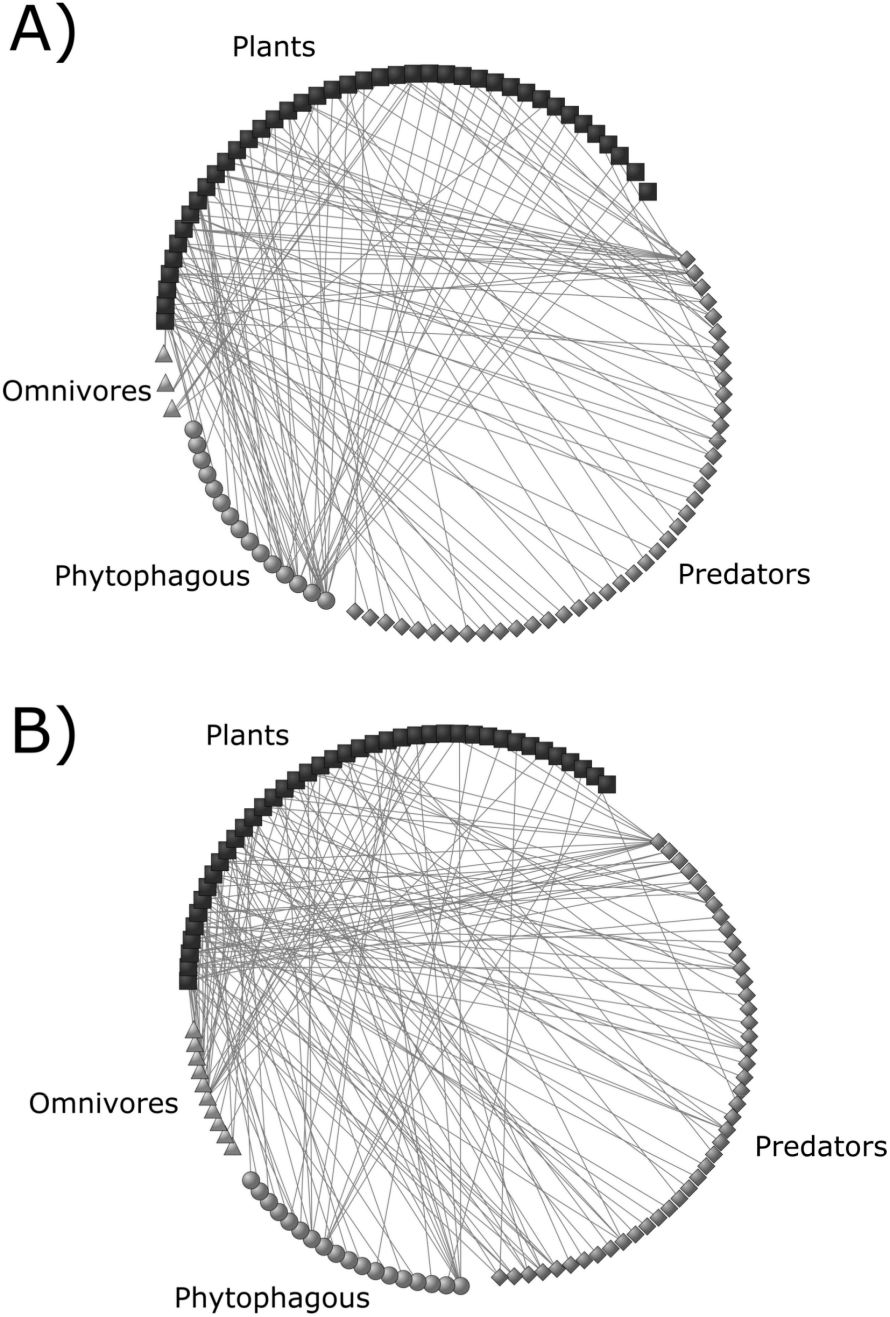
Interaction networks between individuals of *Heliconia aurantiaca* and their arthropod inhabitants divided into three trophic guilds inserted in (A) continuous and (B) forest fragments. Each node represents one animal species or plant individual, and lines represent plant-animal interactions.

**Fig 4:**
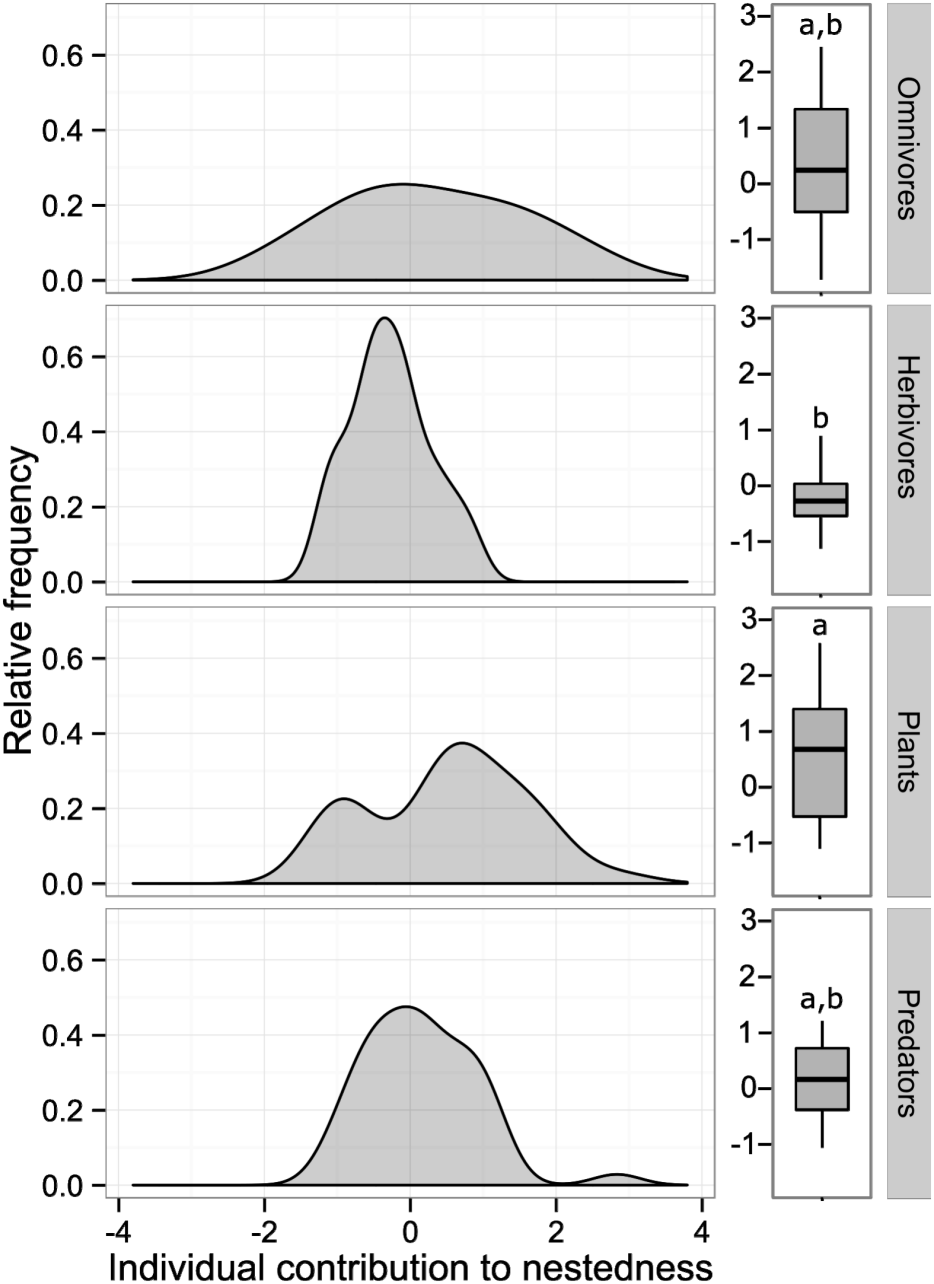
Relative frequency of individual nestedness contribution for all species within each of three trophic guilds. The data on absolute values of individual nestedness is presented as box plots illustrating the median (center line), quartile (box edges), and extreme values (bars) of each trophic guild. Boxplots sharing the same case letters are not significantly different among themselves according to Tukey *post hoc* tests.

By evaluating the patterns of interactions, we found a higher diversity of interactions (*DI* = 451) in the continuous forest compared to forest fragments (*DI* = 422). Interestingly, only in forest fragments the interaction network involving plants and their associated fauna was significantly nested (*NODF* = 8.99, *P* = 0.02) (Fig 3). The interaction network in the continuous forest did not exhibit a nested pattern (*NODF* = 8.79, *P* = 0.19). Contributions to nestedness differed greatly among plant and animal species for the interaction network in fragments (ANOVA: *F*_3, 109_ = 32.73, df = 3, *P* = 0.02), and only a few species within each trophic guild contributed strongly to the nested pattern. In general, plants (mean_*ci*_ ± SE: 0.28 ± 1.03), omnivores (0.27 ± 1.28) and predators (0.15 ± 0.76) contributed more to nestedness than herbivores (-0.27 ± 0.55) (Fig 4).

## Discussion

Our results indicate that forest fragmentation influences the relative density and diversity of arthropods associated with *H*. *aurantiaca* herbs. Species richness remained fairly constant, but there was a marked species turnover in trophic guilds from continuous forest to fragments. Contrary to other findings on arthropod assemblages in conserved and disturbed habitats, we found that the structure of *Heliconia*-invertebrate networks is not maintained in forest fragments [8–10]. The changes in the local abundance, diversity and composition of invertebrates across trophic guilds may seem to be caused by the combined effects of abiotic and biotic factors.

### Habitat fragmentation and the arthropod community

Physical and biological factors in fragments may affect the rates of colonization and extinction of different arthropod taxa and trophic guilds in contrasting ways. In tropical rain forest fragments greater canopy openness tends to favor disturbance-loving insect herbivores (e.g. leaf-cutter ants; [7,36]). In fact we found a greater abundance and richness of ant species associated with *H*. *aurantiaca* in forest fragments, many of which were omnivorous species. The density of ants inhabiting *H*. *aurantiaca* was six times greater in forest fragments than in continuous forest; fragments hold more than twice the number of ant species (17 ant species) than continuous forest (seven ant species). Nevertheless, none of the common leaf-cutter ants *Atta* and *Acromyrmex* (common pests in the Neotropics) were recorded in our surveys (but see [33,37,53]). Apart from ants, our results revealed that fragments have a higher proportion of omnivorous species that together with predatory spiders probably represent novel and opportunistic *Heliconia*-arthropod interactions (Fig 2).

Conversely, the abundance of beetles was greatest in continuous forests (70 beetles) than in forest fragments (39 beetles). The Coleoptera was mainly represented by herbivores in both habitats (63 herbivores *vs*. 7 predators, in continuous forest; and 36 herbivores *vs*. 3 predators in forest fragments). Tortoise beetles (Chrysomelidae) of the genus *Spaethiella* sp. were the most common leaf herbivores in our surveys (which is the first record for Mexico [54]). Although evidence suggests that food and habitat are commonly not limited to chrysomelid populations living on heliconias, we found however that their populations are limited in forest fragments (S1 Table). Because of their role as herbivores and their great species richness elsewhere, the chrysomelids are suggested as indicator taxa for monitoring environmental quality and biodiversity loss in conserved and disturbed ecosystems [55]. Lower density of chrysomelid adults and larvae in forest fragments may result from heavy parasitism and predation and from changes in environmental conditions that make their eggs even more sensitive to desiccation [13,16,17]. The low dispersal ability of these insects could be another possible explanation for lower numbers in fragments [16,17,55,56].

### Host traits and the arthropod community

Host quality and density may affect invertebrate growth and reproduction and therefore plant–invertebrate interactions (Table 1) [11,56,57]. Leaf toughness is likely the best defensive strategy in heliconias as several species in the genus have few secondary compounds [56,58, 59]. In the study area, however, the local density and chemical and physical leaf traits of *H*. *aurantiaca* did not differ significantly between habitats (see Table 1), indicating that bottom-up (i.e. defense and nutrient content of plants) herbivory controls are not evident in our study system. On the other hand, top-down controls (i.e. predators and parasitoids) may be regulating the population dynamics of arthropods and therefore the *Heliconia*–arthropod interactions at some level [7].

This study highlights that the persistence of specialized plant-herbivore interactions (*Heliconia*-chrysomelids) could be limited in forest fragments, in which the understory physical environment, local extinction of some taxa, limited dispersal, reduced reproduction due to an increased predation and parasitism, will likely have deleterious consequences for the colonization of *H*. *aurantiaca* plants by specialized beetles and other insects. The local extinction of species within different trophic guilds may affect the population dynamics of arthropods and therefore herbivory levels. In addition, as herbivores insects act as vectors of disease our results suggest that other biotic interactions may be affected by forest fragmentation as well [15].

### Ecological network patterns in fragments

Nestedness describes the organization of niche breadth within an interactive community in which more nested interactions tend to have the highest niche overlap [9,60,61]. In this study, we found that only in fragments the arthropod community associated with *H*. *aurantiaca* was nested mainly because the increase of specialized herbivorous insects in continuous forest (e.g. *Spaethiella* sp.). Moreover, we observed that more generalized trophic guilds (i.e. plants, omnivores and predators) had a greater contribution to network nestedness than did a trophic guild with specialized species in *Heliconia* (i.e. herbivores). These findings indicate that a *Heliconia*-arthropod network could be more functionally redundant in fragments compared to a *Heliconia*-arthropod network within continuous forest. In other words, habitat fragmentation could be simplifying the community of arthropods interacting with *H*. *aurantiaca* and suggesting novel and/or opportunistic host-arthropod associations as some other interactions may disappear. Additionally, high herbivore diversity can also be promoted if plant–herbivore interactions are specialized, because finely partitioned plant resources will facilitate species coexistence [11]. In fact, fragments exhibited lower diversity of interactions than continuous forest. Therefore, *Heliconia*-arthropod network properties do not appear to be maintained in fragments mainly because of the decrease in specialized herbivorous insects.

## Conclusion

Further studies are still needed to analyze the importance of forest fragmentation in the distribution and phenology of specialized herbivores (e.g. Chrysomelidae) and other arthropods living in *Heliconia* species. Studies including the impact of predators and parasitoids on herbivorous populations, within host heterogeneity between young and old leaves (i.e. insolation, chemical and physical leaf traits), arthropod colonization and spacing of host plants, are necessary to establish the key and regulatory factors in the assemblages of the arthropod communities and trophic guilds inhabiting *Heliconia* species. Regardless, *H*. *aurantiaca* individuals in fragments hold a wide range of arthropods and other invertebrates in different trophic guilds and contribute to the support of arthropod diversity in natural and human-disturbed tropical habitats. Although we have only two replicates per habitat type, the generality of the results is supported by the fact that significant changes in the abundance and diversity of arthropods and trophic guilds occurred from continuous forest to fragments at different sites. However, a larger number of sites per habitat type as well as the replication of the study in other Neotropical forests are needed to strengthen the inference that fragmentation negatively affects arthropod assemblages. The results obtained provide with valuable empiric information on the effects of forest fragmentation on biotic interactions of a particular system (*Heliconia*-arthropod interactions) within the Lacandon rain forest.

## Supporting Information

S1 Table Arthropod species associated to the tropical herb *Heliconia aurantiaca*. Labels according to trophic guilds are: Predators (Pr), Herbivores (Hb), Omnivores (O) and Saprophagous (Sa). Trophic guilds and species are according to Arnett, et al. (2002), Ubick, et al. (2005), Foelix (2011), Groc, et al. (2014). Numbers indicate the abundance of each species within each habitat type, at Lacandon rain forest, Mexico.
(DOC)
