# Supplementary material for: The Multiple Impacts of Tropical Forest Fragmentation on Arthropod Biodiversity and on their Patterns of Interactions with Host Plants
Source: PLoS One. 2016 Jan 5;11(1):e0146461. doi: 10.1371/journal.pone.0146461 (PMC4701723; doi:10.1371/journal.pone.0146461)
Supplement: S1 Table — Labels according to trophic guilds are: Predators (Pr), Herbivores (Hb), Omnivores (O) and Saprophagous (Sa). Trophic guilds and species are according to Arnett, et al. (2002), Ubick, et al. (2005), Foelix (2011), Groc, et al. (2014). Numbers indicate the abundance of each species within each habitat type, at Lacandon rain forest, Mexico. (DOC) [file pone.0146461.s001.doc]

**Supporting information**

S1. Arthropod species associated to the tropical herb *Heliconia aurantiaca*. Labels according to trophic guilds are: Predators (Pr), Herbivores (Hb), Omnivores (O) and Saprophagous (Sa). Trophic guilds and species are according to Arnett, et al. (2002), Ubick, et al. (2005), Foelix (2011), Groc, et al. (2014). Numbers indicate the abundance of each species within each habitat type, at Lacandon rain forest, Mexico.

| Class | Order | Family | Species | Trophic Guild | Continuous Forest | Forest Fragments |
| --- | --- | --- | --- | --- | --- | --- |
| Arachnida | Araneae | Anyphaenidae | Anyphaenidae sp1 | Pr | 1 | 0 |
|  |  | Araneidae | Araneidae sp1 | Pr | 1 | 3 |
|  |  |  | Araneidae sp2 | Pr | 1 | 0 |
|  |  |  | Araneidae sp3 | Pr | 1 | 0 |
|  |  |  | Araneidae sp4 | Pr | 1 | 0 |
|  |  |  | Araneidae sp5 | Pr | 0 | 1 |
|  |  |  | Araneidae sp6 | Pr | 0 | 2 |
|  |  | Clubionidae | Clubionidae sp1 | Pr | 0 | 2 |
|  |  |  | Clubionidae sp2 | Pr | 0 | 1 |
|  |  | Corinnidae | Corinnidae sp1 | Pr | 1 | 0 |
|  |  | Dictynidae | Dictynidae sp1 | Pr | 0 | 1 |
|  |  | Mimetidae | Mimetidae sp1 | Pr | 1 | 0 |
|  |  |  | Mimetidae sp2 | Pr | 2 | 1 |
|  |  | Pholcidae | *Metagonia* sp.1 | Pr | 0 | 2 |
|  |  |  | *Modisimus* sp.1 | Pr | 1 | 0 |
|  |  | Pisauridae | Pisauridae sp1 | Pr | 0 | 1 |
|  |  | Salticidae | *Acragas* sp.1 | Pr | 1 | 0 |
|  |  |  | *Barva* sp.1 | Pr | 1 | 1 |
|  |  |  | *Freya* sp.1 | Pr | 0 | 1 |
|  |  |  | *Helvetia* sp.1 | Pr | 0 | 1 |
|  |  |  | *Hypaeus* sp.1 | Pr | 0 | 1 |
|  |  |  | *Lyssomanes* sp.1 | Pr | 3 | 12 |
|  |  |  | *Lyssomanes* sp.2 | Pr | 1 | 1 |
|  |  |  | *Lyssomanes* sp.3 | Pr | 0 | 1 |
|  |  |  | *Myrmarachne* sp.1 | Pr | 6 | 4 |
|  |  |  | *Myrmarachne* sp.2 | Pr | 1 | 1 |
|  |  |  | *Sidusa* sp.1 | Pr | 1 | 0 |
|  |  |  | *Synemosyna* sp.1 | Pr | 2 | 3 |
|  |  |  | *Zygoballus* sp.1 | Pr | 1 | 0 |
|  |  |  | *Zygoballus* sp.2 | Pr | 2 | 1 |
|  |  | Scytodidae | *Scytodes* sp.*1* | Pr | 2 | 4 |
|  |  |  | *Scytodes* sp*.2* | Pr | 2 | 4 |
|  |  | Theridiidae | Achaearaneae sp1 | Pr | 0 | 1 |
|  |  |  | *Argyrodes* sp1 | Pr | 0 | 1 |
|  |  |  | *Argyrode*s sp2 | Pr | 0 | 1 |
|  |  |  | *Argyrodes* sp3 | Pr | 0 | 1 |
|  |  |  | *Argyrodes* sp.4 | Pr | 0 | 1 |
|  |  |  | *Argyrodes* sp*.*5 | Pr | 1 | 0 |
|  |  |  | *Argyrodes* sp*.*6 | Pr | 1 | 0 |
|  |  |  | *Chrysso* sp1 | Pr | 0 | 1 |
|  |  |  | *Episinus* sp1 | Pr | 1 | 1 |
|  |  |  | *Theridion* sp1 | Pr | 0 | 2 |
|  |  |  | *Theridion* sp2 | Pr | 1 | 1 |
|  |  | Thomisidae | *Misumenoides* sp1 | Pr | 1 | 0 |
|  |  |  | *Misumenoide*s sp2 | Pr | 2 | 0 |
|  |  |  | *Xysticus* sp1 | Pr | 0 | 1 |
|  |  |  | *Xysticus* sp2 | Pr | 1 | 0 |
|  |  | Uloboridae | *Miagrammopes* sp1 | Pr | 2 | 4 |
| Insecta | Coleoptera | Cantharidae | Cantharidae sp. 1 | Pr | 0 | 1 |
|  |  |  | Cantharidae sp. 2 | Pr | 1 | 0 |
|  |  | Carabidae | Carabidae sp. 1 | Pr | 1 | 0 |
|  |  |  | Carabidae sp. 2 | Pr | 1 | 0 |
|  |  | Chrysomelidae | *Capraita* sp. | Hb | 1 | 0 |
|  |  |  | *Colaspis* sp. | Hb | 1 | 0 |
|  |  |  | *Epitrix* sp. | Hb | 1 | 0 |
|  |  |  | *Margaridisa* sp. | Hb | 0 | 1 |
|  |  |  | *Spaethiella* sp. | Hb | 18 | 11 |
|  |  |  | *Stilodes* sp. | Hb | 0 | 1 |
|  |  |  | *Syphrea smithi* | Hb | 1 | 0 |
|  |  |  | *Syphrea* sp. | Hb | 1 | 0 |
|  |  |  | Larvae sp.1 | Hb | 36 | 11 |
|  |  |  | *Triarius* sp. 1 | Pr | 0 | 2 |
|  |  | Cleridae | Cleridae sp. 2 | Pr | 1 | 1 |
|  |  | Coccinellidae | Coccinellidae sp. 1 | Hb | 0 | 1 |
|  |  |  | Coccinellidae sp. 2 | Hb | 0 | 1 |
|  |  | Curculionidae | Curculionidae sp. 1 | Hb | 1 | 0 |
|  |  |  | Curculionidae sp. 2 | Hb | 0 | 1 |
|  |  |  | Curculionidae sp. 3 | Hb | 1 | 0 |
|  |  |  | Curculionidae sp. 4 | Hb | 1 | 0 |
|  |  |  | Otidocephalini sp. 1 | Hb | 0 | 1 |
|  |  |  | Otidocephalini sp. 2 | Hb | 0 | 1 |
|  |  | Elateridae | *Horistonotus* sp. | Hb | 0 | 2 |
|  |  | Erotylidae | Erotylidae sp. 1 | Hb | 0 | 1 |
|  |  | Hydrophilidae | *Laccobius* sp. | Hb | 1 | 0 |
|  |  | Staphylinidae | Staphilinidae sp. 1 | Pr | 3 | 0 |
|  |  |  | Staphilinidae sp. 2 | Pr | 0 | 1 |
|  |  |  | Larvae sp. 2 | Hb | 0 | 2 |
|  |  | Ptinidae | Ptinidae sp. 1 | Sa | 1 | 5 |
|  |  |  | Ptinidae sp. 2 | Sa | 1 | 0 |
|  |  |  | Ptinidae sp. 3 | Sa | 1 | 0 |
|  |  | Hemiptera | Cicadellidae | Hb | 0 | 1 |
|  |  |  | Cicadellidae sp1 | Hb | 0 | 1 |
|  |  | Pentatomidae | Pentatomidae sp.1 | Hb | 13 | 7 |
|  |  | Psyllidae | Psyllidae sp1 | Hb | 1 | 0 |
|  |  |  | Psyllidae sp2 | Hb | 1 | 0 |
|  |  |  | Psyllidae sp3 | Hb | 0 | 1 |
|  |  |  | Psyllidae sp4 | Hb | 0 | 1 |
|  |  | Reduvidae | Emesinae sp1 | Pr | 0 | 1 |
|  | Hymenoptera | Formicidae | *Camponotus novogranadensis* | O | 0 | 2 |
|  |  |  | *Camponotus planatus* | O | 0 | 44 |
|  |  |  | *Camponotus raphaelis* | O | 0 | 1 |
|  |  |  | *Camponotus sanctaefidei* | O | 0 | 2 |
|  |  |  | *Camponotus sericeiventris* | O | 0 | 2 |
|  |  |  | *Camponotus* sp.1 | O | 0 | 2 |
|  |  |  | *Cephalotes minutus* | O | 0 | 4 |
|  |  |  | *Cephalotes multispinosus* | O | 0 | 6 |
|  |  |  | *Dolichoderus lutosus* | O | 12 | 0 |
|  |  |  | *Dolichoderus bispinosus* | O | 18 | 30 |
|  |  |  | *Pachycondya unidentata* | Pr | 9 | 7 |
|  |  |  | *Pheidole* sp*.*1 | O | 55 | 154 |
|  |  |  | *Pseudomyrmex aff oki* | Pr | 0 | 21 |
|  |  |  | *Pseudomyrmex boopis* | Pr | 0 | 21 |
|  |  |  | *Pseudomyrmex gracilis* | Pr | 0 | 5 |
|  |  |  | *Pseudomyrmex simplex* | Pr | 0 | 5 |
|  |  |  | *Pseudomyrmex* sp. 1 | Pr | 10 | 9 |
|  |  |  | *Pseudomyrmex* sp. 2 | Pr | 27 | 12 |
|  |  |  | *Pseudomyrmex* sp. 3 | Pr | 20 | 0 |
|  | Orthoptera | | Gryllidae | Hb | 8 | 5 |
|  |  |  | Gryllidae sp.1 | Hb | 8 | 5 |

Arnett RH, Thomas MC, Skelley P, Frank JH. 2002. *American beetles. Polyphaga: Scarabaeoidea through Curculionoidea*, vol. 2. CRC, Boca Raton, Florida.

Ubick D, Paquin P, Cushing PE, Roth V. 2005. *Spiders of North America: an identification manual*. American Arachnological Society.

Foelix RF. 2011. Biology of spiders. Oxford University Press, UK.

Groc S, Delabie JHC, Fernández F, Leponce M, Orivel J, Silvestre R, Vasconcelos HL, Dejean A. 2014. Leaf–litter ant communities (Hymenoptera: Formicidae) in a pristine Guianese rain-forest: stable functional structure versus high species turnover. *Myrmec. News* 19:43-51.
